# Antimicrobial resistance: the hidden pandemic we cannot ignore

**DOI:** 10.1016/j.eclinm.2026.104053

**Published:** 2026-06-25

**Authors:** eClinicalMedicine

Since the discovery of penicillin in 1928, antimicrobial agents have proven to be a vital pillar of modern medicine and now almost a century later, the efficacy of these antimicrobial agents is challenged by the increasing prevalence of Antimicrobial Resistance (AMR). AMR is the ability of disease-causing organisms like bacteria, viruses, fungi, and parasites to evolve and resist the medications (antimicrobial agents) that are designed to kill them or inhibit their growth. According to the Institute for Health Metrics and Evaluation's (IHME) data, 1.14 million deaths in 2021 were directly caused by AMR and 39 million people are expected to die from AMR between 2025 and 2050. The UK Health Security Agency stated that in 2024, the number of antibiotic-resistant infections in the UK equated to an average of nearly 400 new cases per week and antibiotic-resistant infections were responsible for 2379 deaths in 2024. Although the fight against AMR is not new, AMR has now become an urgent global public health concern and the basis to economic crisis, forecasted to cost the global economy US$3.4 trillion annually by 2050.

At the 79th World Health Assembly, held in Geneva, Switzerland, in May 2026, member states adopted the updated Global Action Plan on Antimicrobial Resistance (2026–36). The revised framework reflects on lessons learnt since the original 2015 plan and aligns with commitments made during the 2024 United Nations High-Level Meeting on AMR. The updated strategy embraces a comprehensive One Health approach, recognising that human health, animal health, agriculture, food systems, and environmental protection are deeply interconnected and places strong emphasis on infection prevention (Water, Sanitation, and Hygiene [WASH], vaccinations, and biosecurity), widespread surveillance, responsible antimicrobial use, sustainable financing, innovation, and global accountability.

Although the occurrence of resistance is a natural process, its emergence and spread is accelerated by human activity, mainly the continued misuse and overuse of antimicrobials in humans, animals, and plants alike. Although AMR is a global issue, low-income and middle-income countries (LMICs) are affected more because of their higher burden of infection, limited healthcare infrastructure, weak regulatory enforcement, and unrestricted access to antibiotics. Public awareness on the issue remains surprisingly poor and the unintended misuse of antimicrobials is common. Countries, especially LMICs, need strong stewardship programmes and AMR awareness campaigns must become more sophisticated, culturally tailored, and continuous. AMR should be discussed not only by infectious disease specialists and doctors but also by pharmacists, teachers, community leaders, and policy makers.

Globally, large quantities of antibiotics are used in livestock production to promote growth or prevent disease in crowded farming conditions and this creates selective pressure that encourages resistant organisms to emerge and spread from animals to susceptible human hosts. Pharmaceutical waste, agricultural runoff, and untreated sewage can release antimicrobial residues and resistant organisms into ecosystems, becoming breeding grounds for resistance. A recent study has shown that climate change also plays a role in increasing AMR, being associated with at least a 10% rise in antimicrobial resistance genes. Agricultural, environmental, and health-care systems need to be involved collectively in AMR policy making.

The pharmaceutical industry faces a defining moment. As per reports, by 2020, of all the global pharmaceutical giants only six now invest in newer antibiotics and in the past 5 years, pharmaceutical projects related to AMR have reduced by more than 35%. Encouragingly, recent breakthroughs show that innovation remains possible. New oral treatments like gepotidacin and zoliflodacin for drug-resistant gonorrhoea, have shown promising results and represent some of the first big advances in decades of antimicrobial research. Pharmaceutical companies must step up by investing in next-generation antibiotics, novel antifungal agents, and bacteriophage therapies. Earlier this year, WHO published three Target Product Profiles for pharmaceutical companies to focus on, developing novel antibiotics for conditions they are most needed in, including bacterial meningitis, ventilator-associated bacterial pneumonia, and septicaemia. Additionally, companies should also invest in developing rapid diagnostic technologies like metagenomic next-generation sequencing (mNGS) to prevent unnecessary empirical therapy and further misuse of available antibiotics. Companies should expand collaborative research with academic institutions and public-health organisations while committing to equitable global access.

Updated data surveillance systems for resistance patterns represent another crucial front that need to be further developed. The WHO's Global Antimicrobial Resistance and Use Surveillance System (GLASS) has expanded considerably, with more than 100 countries contributing their data. The European Antimicrobial Resistance Surveillance Network (EARS-Net) collects AMR data from all over Europe and guides policy decisions. However, large gaps remain in surveillance quality and coverage, particularly in resource-constrained sub-Saharan Africa and south Asian countries, which bear the heaviest burden. Countries should focus innovation and funds to develop surveillance data systems, because without accurate information on resistance patterns, countries are effectively fighting blind.

Looking ahead, the future direction of AMR policies should be proactive rather than reactive. From inappropriate prescribing to inadequate sewage treatment, a range of human activities drive the emergence and spread of antimicrobial resistance, with implications for the management of common infections. The world has an opportunity to preserve one of medicine's greatest achievements: the ability to cure most bacterial infections. The question is whether the world will act before that achievement slips beyond its reach.

